# The importance of sustained compliance with physical distancing during COVID-19 vaccination rollout

**DOI:** 10.1038/s43856-022-00207-3

**Published:** 2022-11-19

**Authors:** Alexandra Teslya, Ganna Rozhnova, Thi Mui Pham, Daphne A. van Wees, Hendrik Nunner, Noortje G. Godijk, Martin Bootsma, Mirjam E. Kretzschmar

**Affiliations:** 1 Julius Center for Health Sciences and Primary Care, University Medical Center Utrecht, Utrecht University, Utrecht, The Netherlands; 2 BioISI—Biosystems & Integrative Sciences Institute, Faculdade de Ciências, Universidade de Lisboa, Lisboa, Portugal; 3 Centre for Complex System Studies (CCSS), Utrecht University, Utrecht, The Netherlands; 4 Department of Sociology / ICS, Utrecht University, Utrecht, The Netherlands; 5 Department of Mathematics, Faculty of Sciences, Utrecht University, Utrecht, The Netherlands

**Keywords:** Viral infection, Epidemiology

## Abstract

**Background:**

Increasing vaccination coverage against SARS-CoV-2 enabled relaxation of lockdowns in many countries in Europe. As the vaccination rollouts progressed, the public health authorities were seeking recommendations on the continuation of physical distancing measures during ongoing vaccination rollouts. Compliance with these measures was declining while more transmissible virus variants have emerged.

**Methods:**

We used a SARS-CoV-2 transmission model to investigate the feedback between compliance, infection incidence, and vaccination coverage. We quantified our findings in terms of cumulative number of new hospitalisations three and six months after the start of vaccination.

**Results:**

Our results suggest that the combination of fast waning compliance in non-vaccinated individuals, low compliance in vaccinated individuals, low vaccine efficacy against infection and more transmissible virus variants may result in a higher cumulative number of new hospitalisations than in a situation without vaccination. These adverse effects can be alleviated by deploying behavioural interventions that should preferably target both vaccinated and non-vaccinated individuals. The choice of the most appropriate intervention depends on vaccination rate and vaccine efficacy against infection.

**Conclusions:**

Supplementary behavioural interventions aiming to boost compliance to physical distancing measures can improve the outcome of vaccination programmes, until vaccination coverage is sufficiently high. For optimal results, these interventions should be selected based on the vaccine efficacy against infection and expected vaccination rate. While we considered the dynamics of SARS-CoV-2, the qualitative effects of the interplay between infectious disease spread and behavior on the outcomes of a vaccination programme can be used as guidance in a future similar pandemic.

## Plain language summary

In many countries, SARS-CoV-2 (COVID-19) vaccination campaigns commenced in the first months of 2021. As vaccination coverage increased, the compliance with physical distancing measures aimed at reducing virus transmission often declined, raising concern for potential overburdening of health systems before a sufficiently large portion of the population was vaccinated. Using a mathematical model of infection transmission which takes into account behaviour of the population, we identify driving factors of increased transmission. Our findings suggest that to maximize the benefits of vaccination campaigns, compliance with physical distancing measures in vaccinated and non-vaccinated groups should be targeted, prioritizing one group over the other depending on the vaccination rate, the efficacy of vaccine in blocking the infection, and the circulating variant of the virus. Ongoing audience-tailored communication by public health professionals could be key in this undertaking.

## Abstract (web summary)

Teslya et al. examine the importance of sustained compliance with physical distancing during COVID-19 vaccination rollout. Using mathematical models, the authors find that waning compliance may contribute to increased hospitalisations in initial phases of the rollout and that behavioural interventions would likely help to control transmission.

## Introduction

In November 2020, more than 8 months after the outbreak of COVID-19 was declared a pandemic by the World Health Organisation^1^, the state of the pandemic in many countries around the world remained dire, with hospitalisations and death tolls mounting. Amid the second wave that started in September 2020^2–4^, more transmissible^5–7^ SARS-CoV-2 variants emerged (e.g., Alpha (B.1.1.7), Beta (B.1.351), and P.1 (Gamma))^8,9^, causing many countries to reinforce physical distancing measures in order to maintain healthcare capacities and to prevent deaths caused by COVID-19. Since then, even more infectious virus variants, Delta (B.1.617.2) and Omicron (B.1.1.529), emerged. Delta became dominant in Europe^10^ in July 2021, and caused new pandemic waves. As of December 2021/January 2022, Omicron was on its way to replace Delta as the dominant SARS-CoV-2 virus variant in Europe^11^ and in the US^12^. These events underscore that the physical distancing measures, while effective in reducing SARS-CoV-2 transmission during the first wave^13–17^, alone are not sufficient to limit SARS-CoV-2 transmission and to eradicate the need for future lockdowns, further measures such as rigorous vaccination campaigns are required.

Fortunately, on the eve of spread of the Alpha variant in Europe, COVID-19 vaccines developed by BioNTech/Pfizer, Moderna, Johnson & Johnson (Janssen), and AstraZeneca were approved by EMA^18^. FDA approved the first three vaccines for use in the US^19^. Thus, hopes for the end of lockdown periods and relaxation of physical distancing measures were fuelled. Phase 3 randomised clinical trials reported promising vaccine efficacies for preventing laboratory-confirmed symptomatic SARS-CoV-2 infection of 62−92%^20–22^. On 29 March 2021 CDC released a report that the Pfizer/BioNTech and Moderna vaccines have at least 80% effectiveness in preventing COVID-19. These findings are consistent with earlier reports that the three vaccines (Pfizer/BioNTech, Moderna and AstraZeneca) have some effectiveness in blocking SARS-CoV-2 transmission^23–25^.

Following the start of the vaccination rollout in countries around the world, data was collected which showed that the approved vaccines have high efficacy in conferring immunity against infection acquisition (80–95% for Pfizer/BioNTech and Moderna^26–28^, and 76% for Johnson & Johnson^29^). These results were estimated from data collected between December 2020 and April 2021 in the USA. During this period, the original variant and the Alpha variant (B.1.1.7) were the dominant circulating variants.

Since then, a new, more infectious variant, Delta (B.1.617.2), has emerged and became dominant until early 2022 in many European countries^10^ and the USA^30^. A study based on data from Israel estimated a reduction of BNT162b2 efficacy for the Delta variant in preventing infection, with efficacy against this variant equal to 64% after two doses^31^. This estimate was supported by another report based on the data in a highly vaccinated health system workforce of California San Diego Health^32^. Thus, the understanding of how the deployment of vaccines can impact transmission of SARS-CoV-2 is complicated by the emergence of the new variants^33–35^. To slow down the appearance rate of antigenically relevant mutations that may escape protection conferred by existing vaccines as well as to reduce the death toll and the burden on healthcare system, a rigorous and global vaccination campaign seems of utmost importance.

From the start, vaccination rollout, however, faced multiple challenges. Public health services were confronted with structural and logistical obstacles (e.g., insufficient supplies, lack of capacity to administer shots^36–38^). Another factor that may affect vaccination rollout is vaccine acceptance^37^. On the eve of vaccination rollout vaccine acceptance varied greatly across countries from 23.6% in Kuwait to 97% in Ecuador^39,40^. Almost a year later, the disparity in vaccine acceptance across continents and even countries on the same continents were reported to be still present^41^. All these challenges in vaccination rollout led to diverging vaccination rates across different countries^42^.

On the other hand, mass vaccination may also have undesirable transient consequences such as reducing compliance with physical distancing measures. For example, there is evidence that in the Netherlands, in the year following the vaccination rollout, the compliance in the general population, and in the vaccinated population specifically, decreased as compared to the epidemic period where vaccines were not available^43^. For vaccinated individuals, this change in the behaviour may happen since following the vaccination event, they perceive COVID-19 to pose a lower risk for them. Moreover, while some individuals get vaccinated to reduce their risks of disease and to limit the transmission in the community, others may do this to gain admittance to public venues. For example, in the Netherlands, many individuals were vaccinated that did not perceive the threat of SARS-CoV-2 infection as high, and instead did it for other reasons, for example, to obtain a QR code (CTB) needed to access public spaces such as restaurants and entertainment venues^43^. On the other hand, the non-vaccinated individuals may also become less compliant with the physical distancing measures, relying on decreased transmission due to the growing vaccination coverage. This is corroborated by the health belief model^44^ which posits that adoption of self-protective measures is motivated by perceived susceptibility to becoming infected, among other factors. This perceived susceptibility changes dynamically with evolving epidemiological situation, as evidenced by data collected in the Netherlands^45^. Hence, compliance may increase as the epidemic grows and decline with increasing vaccination coverage. A number of modelling studies have shown that the feedback between the epidemic dynamics and human behaviour has an important role in the disease transmission^46–48^. In an earlier modelling study^48^, we showed that relaxation of compliance with physical distancing measures beyond a threshold may cause a notable increase in new infections and hospitalisations. This concern is especially relevant at the start of the vaccination campaign, when vaccination coverage is still low.

We developed a socio-epidemiological model for SARS-CoV-2 transmission to investigate the effects of decline of compliance with physical distancing measures on the dynamics of SARS-CoV-2 transmission as vaccine is rolled out in the population. The transmission dynamics is modelled through a susceptible-exposed-infectious-recovered (SEIR) framework. The vaccine works as all-or-nothing conferring perfect protection against infection acquisition to a fraction of susceptible individuals who receive it. The vaccine delivered to individuals in other disease stages has no effect.

We assume that the vaccination rollout takes place during a government-imposed lockdown, whereupon many public venues are closed or operate at a reduced capacity, thus limiting the average number of contacts. Additionally, the government may issue a set of recommendations with respect to physical distancing. Compliance with these recommendations is captured by a reduction in the daily number of contacts relative to the pre-pandemic level of contacts. The non-vaccinated population is divided into individuals who can be more compliant (henceforth referred to as “compliant”) and less compliant (“non-compliant”) to measures. The reduction in contacts is larger for compliant and smaller for non-compliant populations. On the other hand, we assume that vaccinated individuals perceive themselves protected from COVID-19 and therefore, are no longer compelled to comply with physical distancing measures. Thus, they are not affected by the compliance acquisition-loss process and increase their contact rate above that of non-compliant individuals, thereby returning to nearly pre-pandemic level of contacts. Non-vaccinated individuals can move between compliant and non-compliant modes, and the rates of moving depend on the state of the epidemic and on vaccination coverage. Specifically, more individuals become compliant with physical distancing measures as the incidence of SARS-CoV-2 infection cases increases and lose compliance faster as the proportion of vaccinated individuals grows (see Methods).

In this work our goal is to detect qualitative changes in the epidemic outcomes following the rollout of a vaccination programme for different combinations of vaccine efficacy, vaccine uptake rate, and the compliance loss rate by vaccinated and non-vaccinated individuals and subsequently to identify potential scenarios connected to an increase in incidence of new infections and hospitalisations that may occur. Therefore, we did not account for factors such as age structure of the population, regional variation in contacts patterns, and eventual waning of immunity, which are often included in epidemiological models to attain accurate quantitative predictions.

We considered a baseline scenario without vaccination and several vaccination scenarios. To observe the full spectrum of possible scenarios, we sampled vaccination rate on a wide range, which was based on the observations during the first six months of the vaccination rollout in European countries and Israel^42^. Further, we considered scenarios for three types of SARS-CoV-2 variants. The first variant has the transmission potential of the original variant that was dominant in Europe prior to December 2020. The second variant is a more transmissible, Alpha-like variant (B.1.1.7), that spread in many European countries starting November 2020 and became dominant in April 2021^49^. Finally, we also considered the dynamics of a “hyper-contagious” Delta-like variant (B.1.617.2), which, as of August 2021, became the dominant strain in Europe^10^. We investigated the impact of compliance with physical distancing measures on the numbers of infected and hospitalised individuals over the course of the vaccination rollout. We also compared the cumulative numbers of new infections and hospitalisations after three and six months into the vaccination programme to the numbers without vaccination. We tested the robustness of our findings to the values we chose for the initial conditions and parameters by performing multivariate sensitivity analyses. The values for initial conditions and parameters were sampled continuously.

Next, we considered the potential effects of two interventions aimed at improving compliance. The first intervention is targeted at people who have not been vaccinated yet and aims at keeping their compliance with physical distancing at the level of prior to vaccination rollout. The second intervention is targeted at people who have been vaccinated and aims at keeping their contact rates low. We also considered a combined intervention where both interventions are implemented simultaneously.

Finally, we considered the scenario where in the case of a sharp rise in prevalence which may occur due to the decline of compliance with physical distancing measures, the government may impose additional physical distancing rules to reduce SARS-CoV-2 transmission. To wit, in the Netherlands, following a sharp increase in the number of detected infections in June of 2021, the government imposed additional measures which aimed to reduce infection transmission and which were in effect for nearly a month (July 10, 2021 to August 13, 2021)^50^. We have investigated outcomes of the combination of the vaccination rollout with a lockdown which initiates when the prevalence of infectious cases surpasses a threshold.

In this study we use a mathematical model that takes into account behavioural response of a population to epidemic dynamics and vaccination coverage to investigate under which conditions a transient increase of new infections and hospitalisations above the level in no-vaccination scenario can occur. We also aim to identify how this potential increase can be mitigated by means of compliance-improving interventions targeted at different subpopulations. In summary, we gain the following insights: (a) If vaccine efficacy exceeds a threshold, decrease in prevalence is expected to occur almost immediately; (b) If vaccine efficacy is below that value and vaccinated and non-vaccinated individuals relax their compliance with physical distancing measures, an excess in the cumulative number of new infections and hospitalisations may occur; (c) In this case, fast vaccine uptake rate may not be advantageous as, combined with diminished compliance, it may lead to a substantial relative increase in the number of infections; (d) For three variants that we considered (original, an Alpha-like, and a Delta-like), an intervention targeting the non-vaccinated population is effective in reducing the number of infections and hospitalisations below the no compliance-targeted intervention scenario and reduces the minimum value for vaccine efficacy necessary to lower this number below the no-vaccination scenario level. However, this threshold is still high; (e) Compared to the intervention that targets non-vaccinated individuals, the intervention that targets compliance of vaccinated individuals yields better results in terms of reducing the relative excess of the number of new infections and hospitalisations when vaccine efficacy is low and the vaccination rate is fast. This intervention performs better in a long run than in short run; (f) Slow vaccination with a combined compliance-targeting intervention can reduce numbers of infections as compared to the no-interventions scenario. But in order to reduce the number of hospitalisations below the level of the no-vaccination scenario, vaccine efficacy should exceed 60%; fast vaccination with a combined intervention reduces the number of new infections even for lower vaccine efficacy; (g) Strengthening of the lockdown triggered by the rise in prevalence is another intervention that can prevent increase in the cumulative number of new infections. Our results indicate that the initiation threshold for the lockdown can be sufficiently high, thus potentially allowing for shorter periods of the slowing down of the economy.

## Methods

### Model

**Fig. 1:**
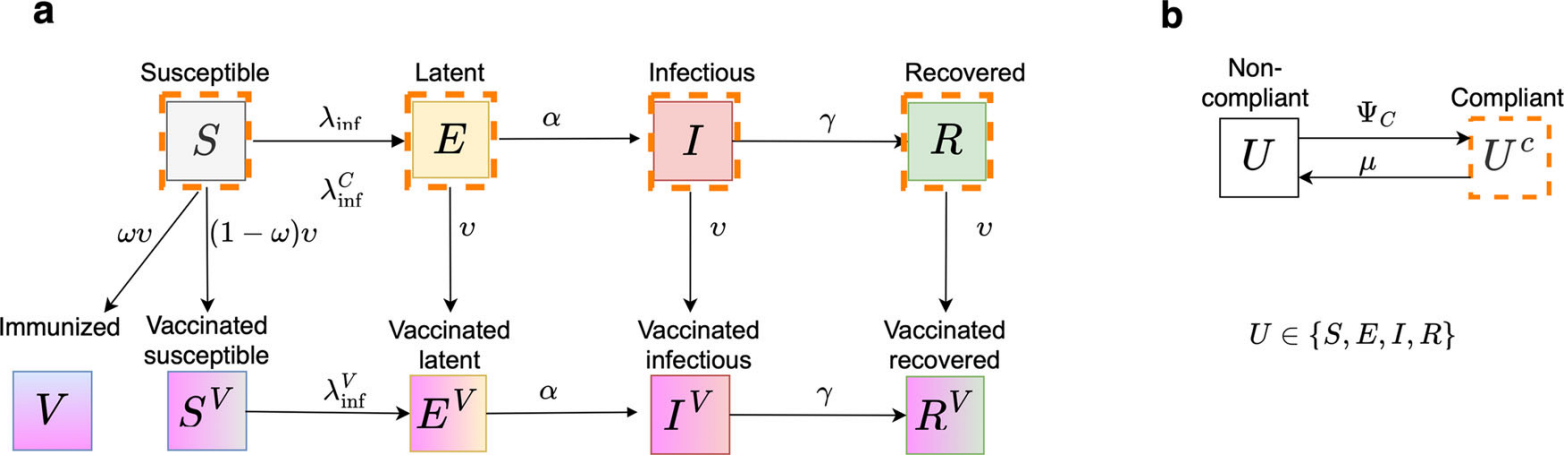
Flow diagram of the infection transmission dynamics coupled with compliance and vaccination processes. **a** Flow diagram of infection transmission and vaccination rollout, **b** Flow diagram of acquisition and loss of compliance. Solid-coloured rectangles denote non-vaccinated compartments; solid-bordered rectangles denote non-compliant compartments; orange dashed-bordered rectangles denote compliant compartments; gradient-coloured rectangles denote vaccinated compartments. Susceptible individuals (*S*, *S*^*C*^, and *S*^*V*^) become exposed (*E*, *E*^*C*^, and *E*^*V*^, respectively) with rates *λ*_inf_, ${\lambda }_{\,{{\mbox{inf}}}}^{C}$, and ${\lambda }_{\,{{\mbox{inf}}}}^{V}$ through contact with infectious individuals (*I*, *I*^*C*^, and *I*^*V*^). Exposed individuals become infectious (*I*, *I*^*C*^, and *I*^*V*^, respectively) at rate *α*. Infectious individuals recover (*R*, *R*^*C*^, and *R*^*V*^) at rate *γ*. Compliance is gained with rate Ψ_*C*_ and lost with rate *μ*. Individuals in any state of infection or compliance can get vaccinated. A proportion *ω* of susceptible individuals *S*, who were vaccinated are fully protected, *V*. Individuals who were vaccinated, but did not obtain protection, are denoted by *S*^*V*^, *E*^*V*^, *I*^*V*^ and *R*^*V*^ and are epidemiologically indistinguishable from their non-vaccinated counterparts.

We developed a compartmental deterministic model that describes SARS-CoV-2 transmission and vaccination rollout in a population. Subsequently, we modified this model to include acquisition and loss of compliance with physical distancing measures as individuals continuously get exposed to information about disease spread as well as about the progress of vaccination rollout (Fig. 1). We informed the model using parameter values from the literature as well as estimating parameters from publicly available data for the Netherlands, Belarus, Denmark, and Israel. We used the model to investigate the effects of interactions between disease transmission, vaccination rollout, and changing compliance with physical distancing measures on transmission dynamics.

#### Population compartments

The SARS-CoV-2 transmission dynamics follow a Susceptible-Exposed-Infectious-Recovered (SEIR) framework that divides the population into the following compartments: susceptible (*S*), latently infected (also referred to as “exposed”, *E*), infectious (*I*), and recovered (*R*). Susceptible individuals (*S*) become latently infected (*E*) with rate *λ*_inf_ proportional to the fraction of infectious individuals (*I*/*N*, where *N* is the total population size). Individuals stay latently infected (*E*) for an average duration of 1/*α* days after which they become infectious (*I*). Infectious individuals recover after an average duration of 1/*γ* days and move to compartment *R*. Because of a relatively short time horizon of our analyses (not exceeding six months) and relatively small case fatality ratio, we disregarded demographic processes such as births and deaths, and therefore the population size *N* is constant. Additionally, we assumed that once individuals recover they acquire permanent immunity and cannot be re-infected. Since we are interested in understanding the qualitative dynamics that follow from interaction of infection transmission, changes in compliance, and vaccination rollout, we did not consider different outcomes of infection with SARS-CoV-2 (e.g., asymptomatic or symptomatic infection, hospitalisation, death etc.). The infectious compartment (*I*), therefore, contains individuals who are asymptomatic, or have mild or severe symptoms.

The dynamics of infection transmission are modelled for three variants of the SARS-CoV-2 virus: first, the original variant that was predominant in Europe prior to April 2021; second, the more transmissible Alpha (B.1.1.7) variant, that was initially detected in the UK and became dominant in many European countries in April of 2021; and finally, the “hyper-contagious” Delta (B.1.617.2) variant, which was dominating in many European countries in July 2021^49^. We parameterized the differences between these variants by using different probabilities of transmission per contact, *ϵ*. We assumed that in all other respects the variants have the same properties. We investigated model dynamics where only one of the three variants circulates in the population.

To model vaccination, the population was stratified into vaccinated and non-vaccinated classes. While for some vaccines authorised for use in Europe (BioNTech/Pfizer, Moderna and AstraZeneca,^18^), two vaccine doses, as well as a certain time period passing after the second dose are required for full immunisation, we modelled vaccination as a single event that confers protection instantaneously. We assumed that individuals do not obtain a diagnostic or antibody test prior to vaccination, and therefore infected and recovered individuals also get vaccinated. Thus, individuals in all epidemiological compartments can get vaccinated, but only those who were susceptible (*S*) at the time of vaccination may become immunised (*V*). The vaccination rate is denoted by *υ*. We assumed that the vaccine works as all-or-nothing, i.e. upon vaccination, a proportion *ω* of susceptible individuals (*S*) is fully protected (*V*), while in a proportion 1 − *ω* of susceptible individuals the vaccine has no effect. We refer to *ω* as “vaccine efficacy” in the context of conferring sterilising immunity. Vaccination does not confer protection to individuals, who were in other infection compartments (*E*, *I* and *R*) at the time of vaccination, and their infection progression is identical to that of non-vaccinated individuals. Individuals who were vaccinated but did not obtain the protection are denoted by *S*^*V*^, *E*^*V*^, *I*^*V*^ and *R*^*V*^.

Studies based on data collected in Israel estimated that the BNT162b2 mRNA COVID-19 vaccine developed by Pfizer/BioNTech reduced the acquisition rate for asymptomatic SARS-CoV-2 infection by 80%^26^ up to 95%^27^. Similarly high efficacy against infection acquisition were reported for the mRNA-1273 COVID-19 vaccine developed by Moderna, NIAID^28^. For the adenovirus Ad26.COV2.S COVID-19 vaccine developed by Janssen Pharmaceutical Companies the efficacy in preventing infection with SARS-CoV-2 was reported to be 76%^29^. These results were estimated from data collected between December 2020 and April 2021 in the USA. During this period, the original variant and the Alpha variant (B.1.1.7) were the dominant circulating variants. Since then, the “hyper-contagious” Delta variant (B.1.617.2) has become dominant in many European countries^10^ and the USA^30^. A study based on data from Israel estimated a noteworthy reduction of BNT162b2 efficacy for the Delta variant in preventing infection, which was 64% after two doses^31^. This estimate was supported by another report based on the data in a highly vaccinated health system workforce of California San Diego Health^32^. Therefore, in our analyses, we varied *ω* in the range of 0.4 and 1.0.

Finally, in addition to infection status, unvaccinated individuals in the model are either compliant or non-compliant with physical distancing measures (compliant compartments denoted by superscript *C*: *S*^*C*^, *E*^*C*^, *I*^*C*^, and *R*^*C*^). Compliant individuals thus have on average a lower contact rate than non-compliant individuals; both contact rates are assumed to be lower than pre-pandemic levels. We denote the average contact rate of non-compliant individuals by *c*, and define a reduction factor *r*_1_ that describes the reduction in contact rate of compliant individuals compared to non-compliant individuals, so 0 ≤ *r*_1_ ≤ 1. Transitions between the compliant and non-compliant state are described by a modelling framework similar to Perra et al^46^ and previously used in^48^. We modelled the compliance acquisition rate, Ψ_*C*_, as a function of the incidence of infection, assuming that individuals obtain information about numbers of cases through mass-media and health authorities. We assumed that compliance wanes when case numbers drop or when the disease is no longer present, and individuals return to the non-compliant state at rate *μ*. If there is no-vaccination programme in place then this rate, *μ*, is constant. However, if vaccination rollout is in progress, as vaccination coverage increases, individuals may feel less motivated to comply with physical distancing measures; we implemented this effect by taking *μ* as a linear function of vaccination coverage, i.e. the rate of losing compliance increases with increasing vaccination coverage. We assumed that only non-vaccinated individuals can be in the compliant state, while vaccinated individuals move into a separate non-compliant state permanently, and even have higher contact rates than non-vaccinated non-compliant individuals. We use *r*_2_≥1 to denote the increase in the contact rates of vaccinated individuals relative to the contact rate of non-compliant individuals, *c*. Compliant individuals get vaccinated at the same rate as non-compliant individuals. All individuals who were vaccinated will have the same (increased) contact rate regardless of whether vaccination was successful.

#### Rates

In this section we define the transition rates that depend on the incidence of infectious cases and on vaccination coverage: rates of infection acquisition, and rates of acquisition and loss of compliance.

We assumed that individuals become infected at a rate that depends on the fractions of different types of infectious individuals, as well as on the mixing of compliant, non-compliant and vaccinated individuals. Therefore, infection acquisition rates as well as infection transmission rates depend on compliance and vaccination status of susceptible and infectious individuals. We define the following matrix to summarize transmission rates between different types of susceptible and infectious individuals.

1 $$M=\frac{c\epsilon }{N(t)+{r}_{1}{N}^{C}(t)+{r}_{2}{N}^{V}(t)}\left[\begin{array}{ccc}1&{r}_{1}&{r}_{2}\\ {r}_{1}&{r}_{1}^{2}&{r}_{1}{r}_{2}\\ {r}_{2}&{r}_{1}{r}_{2}&{r}_{2}^{2}\end{array}\right]$$

with

$$N(t) =S(t)+E(t)+I(t)+R(t)\\ {N}^{C}(t) ={S}^{C}(t)+{E}^{C}(t)+{I}^{C}(t)+{R}^{C}(t)\\ {N}^{V}(t) =V(t)+{S}^{V}+{E}^{V}(t)+{I}^{V}(t)+{R}^{V}(t),$$

where [*M*]_11_ captures the transmission of infection from non-compliant *I* to non-compliant *S*, [*M*]_12_ from compliant *I* to non-compliant *S*, and [*M*]_13_ from vaccinated *I* to non-compliant *S*. Similarly, the second row of the matrix captures the transmission of infection to susceptible individuals who are compliant, *S*^*C*^. Finally, the third row of the matrix captures the transmission of infection to individuals who are susceptible despite vaccination, *S*^*V*^. For the derivation of matrix *M* given by equation (1) see Supplementary methods.

We assumed that as individuals learn about new infections they become compliant with physical distancing measures, and therefore compliance is gained at a rate Ψ_*C*_ which is a positive increasing function of the incidence of infectious cases (equal to the rate with which individuals leave the exposed stage):

2 $${\Psi }_{C}(t)=\delta \cdot \alpha \cdot \left[E(t)+{E}^{C}(t)+{E}^{V}(t)\right].$$

We assumed that compliance is not permanent, becoming shorter as the vaccination coverage grows, and thus we model compliant state to have an average duration 1/*μ*, such that *μ* is a positive increasing function of the vaccination coverage, $\bar{V}(t)/N$:

3 $$\mu (t)={\mu }_{0}+{\mu }_{1}\bar{V}(t)/N.$$

#### Equations

The system of ordinary differential equations (4) provides a full description of the model.

Dynamics of non-compliant individuals:

$$\frac{{{{{{{{\rm{d}}}}}}}}S(t)}{{{{{{{{\rm{d}}}}}}}}t}=	 -{\lambda }_{{{\mbox{inf}}}}(t)S(t)-{\Psi }_{C}(t)S(t)+\mu (t){S}^{C}(t)-\upsilon S(t)\\ \frac{{{{{{{{\rm{d}}}}}}}}E(t)}{{{{{{{{\rm{d}}}}}}}}t}=	 \;{\lambda }_{{{\mbox{inf}}}}(t)S(t)-\alpha E(t)-{\Psi }_{C}(t)E(t)+\mu (t){E}^{C}(t)\\ 	-\upsilon E(t)\\ \frac{{{{{{{{\rm{d}}}}}}}}I(t)}{{{{{{{{\rm{d}}}}}}}}t}=	 \;\alpha E(t)-\gamma I(t)-{\Psi }_{C}(t)I(t)+\mu (t){I}^{C}(t)-\upsilon I(t)\\ \frac{{{{{{{{\rm{d}}}}}}}}R(t)}{{{{{{{{\rm{d}}}}}}}}t}=	 \;\gamma I(t)-{\Psi }_{C}(t)R(t)+\mu (t){R}^{C}(t)-\upsilon R(t)$$

Dynamics of compliant individuals

4 $$\frac{{{{{{{{\rm{d}}}}}}}}{S}^{C}(t)}{{{{{{{{\rm{d}}}}}}}}t}=	 \;-{\lambda }_{\,{{\mbox{inf}}}}^{C}(t){S}^{C}(t)+{\Psi }_{C}(t)S(t)-\mu (t){S}^{C}(t)-\upsilon {S}^{C}(t)\\ \frac{{{{{{{{\rm{d}}}}}}}}{E}^{C}(t)}{{{{{{{{\rm{d}}}}}}}}t}=	 \;{\lambda }_{\,{{\mbox{inf}}}}^{C}(t){S}^{C}(t)-\alpha {E}^{C}(t)+{\Psi }_{C}(t)E(t)-\mu (t){E}^{C}(t)\\ 	-\upsilon {E}^{C}(t)\\ \frac{{{{{{{{\rm{d}}}}}}}}{I}^{C}(t)}{{{{{{{{\rm{d}}}}}}}}t}=	 \;\alpha {E}^{C}(t)-\gamma {I}^{C}(t)+{\Psi }_{C}(t)I(t)-\mu (t){I}^{C}(t)\\ 	 -\upsilon {I}^{C}(t)\\ \frac{{{{{{{{\rm{d}}}}}}}}{R}^{C}(t)}{{{{{{{{\rm{d}}}}}}}}t}=	 \;\gamma {I}^{C}(t)+{\Psi }_{C}(t)R(t)-\mu (t){R}^{C}(t)-\upsilon {R}^{C}(t)$$

Dynamics of vaccinated individuals:

$$\frac{{{{{{{{\rm{d}}}}}}}}V(t)}{{{{{{{{\rm{d}}}}}}}}t}= 	\;\omega \upsilon \left(S(t)+{S}^{C}(t)\right)\\ \frac{{{{{{{{\rm{d}}}}}}}}{S}^{V}(t)}{{{{{{{{\rm{d}}}}}}}}t}= 	\;(1-\omega )\upsilon \left(S(t)+{S}^{C}(t)\right)-{\lambda }_{\,{{\mbox{inf}}}}^{V}(t){S}^{V}(t)\\ \frac{{{{{{{{\rm{d}}}}}}}}{E}^{V}(t)}{{{{{{{{\rm{d}}}}}}}}t}=	 \;{\lambda }_{\,{{\mbox{inf}}}}^{V}(t){S}^{V}(t)+\upsilon \left(E(t)+{E}^{C}(t)\right)-\alpha {E}^{V}(t)\\ \frac{{{{{{{{\rm{d}}}}}}}}{I}^{V}(t)}{{{{{{{{\rm{d}}}}}}}}t}=	 \;\alpha {E}^{V}(t)+\upsilon \left(I(t)+{I}^{C}(t)\right)-\gamma {I}^{V}(t)\\ \frac{{{{{{{{\rm{d}}}}}}}}{R}^{V}(t)}{{{{{{{{\rm{d}}}}}}}}t}=	 \;\gamma {I}^{V}(t)+\upsilon \left(R(t)+{R}^{C}(t)\right)\\ \frac{{{{{{{{\rm{d}}}}}}}}\bar{V}(t)}{{{{{{{{\rm{d}}}}}}}}t}= 	\;\upsilon \left(S(t)+E(t)+R(t)+{S}^{C}(t)+{E}^{C}(t)+{R}^{C}(t)\right)\\ 	+\upsilon \left(I(t)+{I}^{C}(t)\right),$$

where

5a $${\lambda }_{{{\mbox{inf}}}}(t)={[M(t)]}_{11}I(t)+{[M(t)]}_{12}{I}^{C}(t)+{[M(t)]}_{13}{I}^{V}(t)$$

5b $${\lambda }_{\,{{\mbox{inf}}}}^{C}(t)={[M(t)]}_{21}I(t)+{[M(t)]}_{22}{I}^{C}(t)+{[M(t)]}_{23}{I}^{V}(t)$$

5c $${\lambda }_{\,{{\mbox{inf}}}}^{V}(t)={[M(t)]}_{31}I(t)+{[M(t)]}_{32}{I}^{C}(t)+{[M(t)]}_{33}{I}^{V}(t).$$

### Parameters and initial data

A full list of parameters and their values is given in Table 1. Here we elaborate on our choice of initial conditions, as well as on the chosen values of the behavioural parameters.

**Table 1:** Summary of model parameters.

Name	Description (unit)	Value^*^	Source
Epidemiological parameters			
*R*_0_	Basic reproduction number, original variant	2.5	^54,55^
${R}_{0}^{{{{{{{{\rm{new}}}}}}}}}$	Basic reproduction number, Alpha (B.1.1.7)-like variant	3.75	^6,7^
${R}_{0}^{{{{{{{{\rm{new}}}}}}}}}$	Basic reproduction number, Delta (B.1.617.2)-like variant	4.92	^61^
*R*_*e*_	Effective reproduction number, original variant	1.1	Computed using the method in^60^
$\hat{c}$	Average contact rate prior to the epidemic (individuals/day)	14.9	^58^
*ϵ*	Probability of transmission per contact, original variant	2.4 × 10^−2^	From ${R}_{0}=\hat{c}\epsilon /\gamma =2.5$
*ϵ*^Alpha^	Probability of transmission per contact, Alpha-like variant	3.6 × 10^−2^	From ${R}_{0}=\hat{c}{\epsilon }^{Alpha}/\gamma =3.75$
*ϵ*^Delta^	Probability of transmission per contact, Delta-like variant	5.4 × 10^−2^	From ${R}_{0}=\hat{c}{\epsilon }^{Delta}/\gamma =4.92$
*c*	Average contact rate of non-compliant individuals starting November 16, 2020 (individuals/day)	8.8 (0.5 − 15)	Obtained from solving *R*_*e*_(0) = 1.1
*r*_1_	Ratio between contact rates of compliant and non-compliant individuals	0.34 (0.01 − 1)	Assumed, control parameter
*r*_2_	Ratio between contact rates of vaccinated and non-compliant individuals	1.5 (1, 1.5)	Assumed, control parameter
1/*α*	Average duration of latent period (days)	4 (2–6)	^54–56^
1/*γ*	Average duration of infectious period (days)	7 (5–9)	^53^
Compliance parameters			
*δ*	Rate of moving to compliant state (1/day)	4 × 10^−5^ (10^−6^ − 10^−4^)	Assumed, control parameter
1/*μ*_0_	Average duration of compliant state when there is no vaccination (days)	30 (7 − 30)	Sensitivity analyses
*μ*_1_	Parameter describing how loss of compliance increases depending on vaccination coverage (1/day)	0, 0.3	Sensitivity analyses
Vaccination parameters			
*υ*	Vaccine uptake rate (1/day)	(5, 60) × 10^−3^	Based on vaccination data in^42^
*ω*	Vaccine efficacy in conferring protection against becoming infected	0.6 (0.55 − 0.95)	^24–27,29,31,32,80,82^, control parameter
Lockdown parameters			
	Threshold of infectious individuals for strengthening/relaxation of the lockdown (individuals)	50 − 500	Sensitivity analysis
	Average contact rate during strengthened lockdown (individuals/day)	3	Sensitivity analysis

^*^Interval was used in sensitivity analyses.

#### Initial data

To model the transmission dynamics of SARS-CoV-2 virus, the model was calibrated to the state of the epidemic and the level of compliance with physical distancing measures prior to the start of vaccination in the Netherlands in November 2020. We used the approximation made by RIVM for the week November 11–17 for the number of infectious individuals and set the total number of currently infectious individuals, *I* + *I*^*C*^, at the start of vaccination rollout to 112,435^4^. We have used this value in the main analysis and performed sensitivity analysis to investigate the sensitivity of our results to this choice (Supplementary notes, Supplementary Fig. 12).

The size of the population that recovered from SARS-CoV-2 infection, *R* + *R*_*C*_, was set based on seroprevalence data from the serological study in an age-stratified and regionally weighted representative sample of the Dutch population^51,52^. The estimated seroprevalence was 4% in June/July of 2020^51^ and increased to 14% in February 2021^52^. To account for the effects of the second wave until the start of vaccination (taken in the simulations to be November 2020) we fixed the recovered population at 8%, and performed sensitivity analysis with respect to this initial value (Supplementary notes, Supplementary Fig. 11).

To estimate the total number of exposed individuals, *E* + *E*^*C*^ at the start of the vaccination rollout, we assumed that, at the time, the epidemiological dynamics are in (pseudo) equilibrium, with the prevalence of infectious cases equal to 112,435 individuals^4^ which was approximated by RIVM using hospital admissions and data from the Pienter Corona study^52^ in the period used for the model calibration. Using the average duration of infectious period equal to 7 days^53^, we estimated that, at the start of the vaccination rollout, the daily incidence of new cases was 16,062 individuals. Using the average duration of the exposed period of infection equal to 4 days^54–56^, we obtained *E* + *E*^*C*^. Having fixed the size of susceptible (*S* + *S*^*C*^), exposed (*E* + *E*^*C*^), and recovered (*R* + *R*^*C*^) compartments and using the total population size of the Netherlands, the size of the susceptible compartment (*S* + *S*^*C*^) follows. We performed sensitivity analysis with respect to the initial value of exposed individuals (Supplementary notes, Supplementary Fig. 13).

We have set the initial proportion of compliant individuals to 65%. This was based on data on the compliance with maintaining a distance of 1.5m, from a study on behavioural measures and well-being conducted between November 11–15, 2020^57^ in the Netherlands. We have investigated the sensitivity of the outcomes that we collected to this value (Supplementary notes, Supplementary Fig. 10).

We obtain

6 $$\frac{S}{S+{S}^{C}}=\frac{E}{E+{E}^{C}}=\frac{I}{I+{I}^{C}}=\frac{R}{R+{R}^{C}}$$

Using Eq. (6) and the percentage of compliant population, initial values for *S*, *E*, *I*, *R*, *S*^*C*^, *E*^*C*^, *I*^*C*^, *R*^*C*^ follow.

Setting the total population size to be equal to approximately that of the Netherlands, 1.7 × 10^7^ we obtain the initial data:

$$S(0)= \;5,412,160,\,E(0)=22,487,\,I(0)=39,352,\\ R(0)= \;476,000,\,{S}^{C}(0)=10,051,156,\,{E}^{C}(0)=41,762,\\ {I}^{C}(0)= \;73,082,\,{R}^{C}(0)=884,000.$$

The initial values for the remaining compartments are set to 0.

#### Contact rates

We defined a contact as an encounter with another individual that is sufficiently long to have a conversation, or that involves physical interactions^58^. The pre-pandemic contact rate in the Netherlands was reported to be equal to 14.9 individuals per day^58^. We assume that the population is in the state of a partial lockdown at the start and throughout the vaccination rollout. In addition to the lockdown-related changes in the contact rate, individuals may reduce the contact rate further by complying with government-recommended physical distancing measures (e.g. work from home as much as possible). A fraction of the population is more compliant with these physical distancing measures and the remaining fraction is less compliant, such that contact rates in the compliant and non-compliant states are constant and the average contact rate is lower than pre-pandemic contact rate. However, as a consequence of vaccination and subsequent loss of compliance the average contact rate in the total population will change in time.

We fixed the contact rates for compliant and non-compliant individuals such that the effective reproduction number *R*_*e*_ at the start of the vaccination rollout was 1.1 (Supplementary methods, Supplementary Fig. 1), which is in agreement with the estimate of *R*_*e*_ reported for the Netherlands in November 2020^59^. We calculated *R*_*e*_(0) assuming that the basic reproduction for the original variant is ${R}_{0}=\beta /\gamma =\hat{c}\epsilon /\gamma =2.5$^54,55^.

Recall that the contact rates of non-compliant and compliant individuals are denoted by *c* and *r*_1_*c*. We calculated the effective reproduction number using the method described in^60^ as

7 $${R}_{e}=\frac{\epsilon cS(0)}{\gamma (N(0)+{N}^{c}(0){r}_{1})}+\frac{\epsilon {r}_{1}c{S}_{c}(0)\left({\mu }_{0}(\alpha +\gamma +{\mu }_{0})+\alpha \gamma {r}_{1}\right)}{\gamma (\alpha +{\mu }_{0})(\gamma +{\mu }_{0})(N(0)+{N}^{c}(0){r}_{1})}.$$

The value *R*_*e*_ = 1.1 is obtained for pairs of contact rates of non-compliant individuals, *c*, and compliant individuals, *r*_1_*c* (see Supplementary methods, Supplementary Fig. 1).

Of all pairs of contact rates that satisfy *R*_*e*_(0) = 1.1, we selected a combination such that the weighted average contact rate for the population at the start of the vaccination is 5 contacts per day. This value exceeds the reported number of contacts in the Netherlands during the government-imposed physical distancing measures in March 2020 by 1.5 contacts but is lower than the reported contact rate of 8.8 per day that was observed in June 2020, when some of the physical distancing measures were relaxed^58^. The chosen parameter pair is *c* = 8.8 and *r*_1_*c* = 2.8. We investigated sensitivity of our outputs to the selected values of the contact rates of compliant and non-compliant individuals (Supplementary notes, Supplementary Figs. 16 and 17).

Contact rates of vaccinated individuals were taken to be 1.5 times the contact rate of non-compliant individuals, assuming that after vaccination individuals will nearly return to the pre-pandemic contact behaviour^58^.

#### Virus variants

We have assumed that the baseline epidemiological dynamics when each one of the three variants circulate are identical, except for the probability of transmission per contact. Thus, the effective reproduction number for the Alpha-like variant was 1.65, i.e. 50% higher than for the original variant^6^. We have set the basic reproduction number for the Delta-like variant using the estimate of 4.92^61^, which makes it ~2 times more transmissible as the original variant. Therefore, the effective reproduction number for the Delta-like variant was approximately equal to 2.2 at the start of the vaccination rollout.

#### Vaccination

**Fig. 2:**
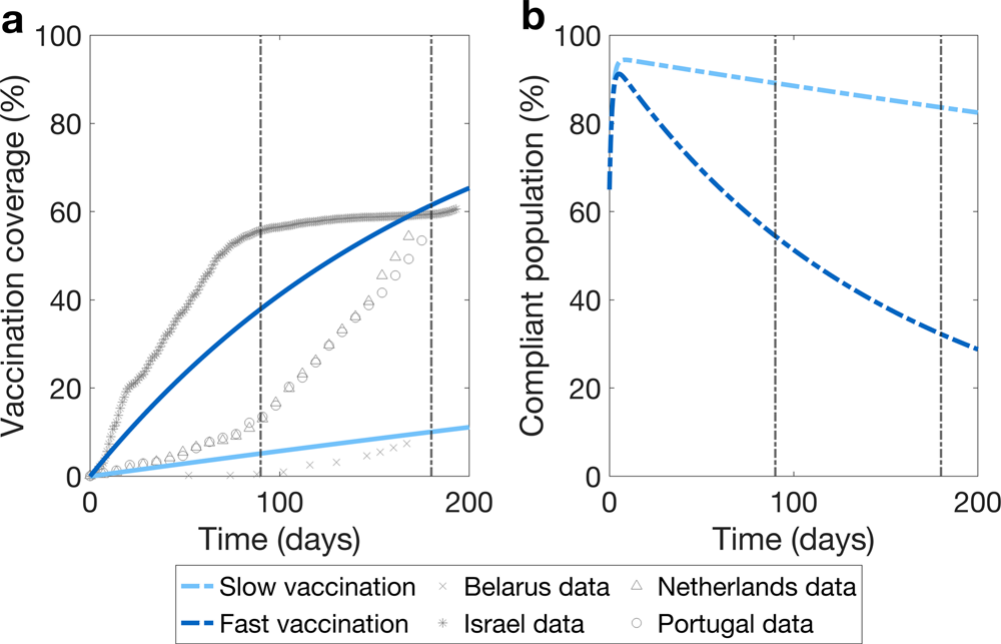
Vaccination coverage and proportion of compliant population during the vaccination rollout. **a** Increase in vaccination coverage for slow (light blue line) and fast (dark blue line) vaccination rates. Light grey markers show vaccination coverage for Belarus (crosses), Israel (stars), Netherlands (triangles), and Portugal (circles), respectively^42^.Smooth lines are output curves of the model indicating the proportion of the total population vaccinated if the whole population was eligible for vaccination and was vaccinated at a rate calculated using these data points. **b** Decrease in the proportion of who complies with physical distancing measures for slow and fast vaccination and a fixed incidence of infection (16,062 cases per day) observed in the Netherlands in the period used for the model calibration. Vertical brown lines mark three and six months since the start of vaccination.

The vaccination rates were sampled on an interval. The lower boundary of the interval is based on the data from the first 6 months of vaccination rollout in Belarus^42,62,63^, one of the slower vaccinating countries in Europe at the time, and would lead to 10% of the total population to be vaccinated in the first 6 months of the vaccination rollout. The upper boundary of the vaccination rate interval is based on the vaccination rollout in Israel in the first 6 months of the vaccination campaign^42,62,63^. Sustaining this rate would lead to almost 60% of the total population to be vaccinated 6 months after the start of the vaccination rollout. Observe that, in real life, at the time only these who were ages 16 years and up were vaccinated. To estimate the boundaries of the vaccination rate interval, we applied linear regression to the data from the respective countries at the start of the vaccination rollout^42,62,63^. Henceforth, these rates are referred to as “slow” and “fast”, respectively. We provide the code as a part of the model suite that we developed to perform the analyses presented in this work^64^. Figure 2a shows vaccination coverage during the first 6 months after the start of vaccination rollout for slow and fast vaccination, as well as the data points for vaccination coverage growth in Belarus, Israel, the Netherlands, and Portugal. In our analyses, we considered a wide range of vaccine efficacies with respect to prevention acquisition of the infection, 55% to 95%. For some of the results in the main analysis we have fixed the vaccine efficacy to 60% and 91%, however subsequently we explored sensitivity of the outcomes to this parameter in an exhaustive fashion.

#### Compliance

In our model, individuals become compliant if there are infectious individuals in the population, such that the per capita rate of switching to the compliant state is proportional to the incidence of infectious cases (see Eq. (2), Table 1). The proportion of compliant and non-compliant individuals in the population is determined by the compliance acquisition rate *δ* and compliance loss rate *μ*. For the main analysis we fixed the duration of compliance when there is no vaccination, 1/*μ*_0_ to 30 days.

We selected the per capita rate of moving to the compliant state, *δ* = 4 × 10^−5^ so that given a constant daily incidence of 16,062 cases, in the regime where the epidemic is seeded with the original variant in a population without any physical measures, 95% of the population is expected to be compliant. This value denotes the case with high compliance acquisition rate. We investigated the sensitivity of the outputs to variation in per capita rate of moving to the compliant state and the compliance loss rate (Supplementary notes, Supplementary Figs. 18 and 19).

In the main analysis we considered a compliance decay scenario where as the vaccination coverage grows, the average duration of compliance decreases (Eq. (3)), in particular, when 33% of the population is vaccinated the compliant state lasts on average 7 days.

The proportion of compliant population for a constant daily incidence of 16,062 cases, which we used to initialize the model is shown in Fig. 2b where we used slow and fast vaccination rates from Fig. 2a. For slow vaccination, three months after the start of vaccination, ~89% of the population is compliant with physical distancing measures and after six months, 84% is compliant. For fast vaccination, the compliant population decreases more rapidly, with only ~54% and 32% of individuals being compliant after three and six months, respectively. These dynamics occur when the growth rate of compliant decay rate as the vaccination coverage increase is *μ*_1_ = 0.3 per day.

The reason for the decline of compliance observed in Fig. 2b is two-fold. First, as the vaccination coverage increases, the compliance in the non-vaccinated population decreases. Moreover, the speed of this decrease depends on how fast vaccination is rolled out. Second, per our assumption, vaccinated people perceiving themselves protected from COVID-19, subsequently comply less with physical distancing. These two processes translate into varying proportions of the compliant population depending on both the incidence of infection and vaccination coverage.

#### Hospitalisation rates

We estimated hospitalisation rates for the original variant, an Alpha-like variant and a Delta-like variant with and without the vaccination. For the original variant we have used estimations of proportions of symptomatic cases and proportion of hospitalised individuals among the symptomatic cases calculated in^65^ and^66^. Subsequently, we have arrived at the value of 4%. To estimate the hospitalisation rate for an Alpha-like variant we have used the note on the severity of B.1.1.7 prepared by New and Emerging Respiratory Virus Threats Advisory Group (NERVTAG) on SARS-CoV-2 variant B.1.1.7^67^. This note estimated the increase in the hospitalisation rate due to B.1.1.7 variant infection between 1.36 to 1.63 times. We used the value of 1.4 to arrive at 5.6%. To calculate the probability of hospitalisation for individuals infected with the Delta variant, we used analysis in^68^ which estimated increase of 1.85 times in the risk of hospitalisation as compared to the Alpha variant. Thus, we obtain 10%. To obtain the hospitalisation rates for the vaccinated individuals we used vaccine efficacy in preventing hospitalisation 95% for the original variant and analysis in^69^, which estimated vaccine efficacy in preventing hospitalisation against the Delta and Alpha variant to be 85%. Respective hospitalisation probabilities are 0.2%, 0.84% and 1.55%. These parameter values are fixed throughout all analyses.

Finally, we investigated the sensitivity of our outputs to the selected average durations of the latent and infectious periods. The analysis can be found in Supplementary notes, Supplementary Figs. 14 and 15.

### Reporting summary

Further information on research design is available in the Nature Portfolio Reporting Summary linked to this article.

## Results

### Epidemic dynamics with vaccination

To quantify the effects of the feedback between epidemic dynamics, vaccination coverage and compliance we use the following outputs of the model: prevalence of currently active infections (exposed and infectious), prevalence of currently hospitalised individuals and the difference in the cumulative number of new hospitalisations relative to the no-vaccination scenario level three and 6 months after the start of the vaccination rollout. We have calculated the prevalence of hospitalised individuals as a proportion of currently infectious cases.

**Fig. 3:**
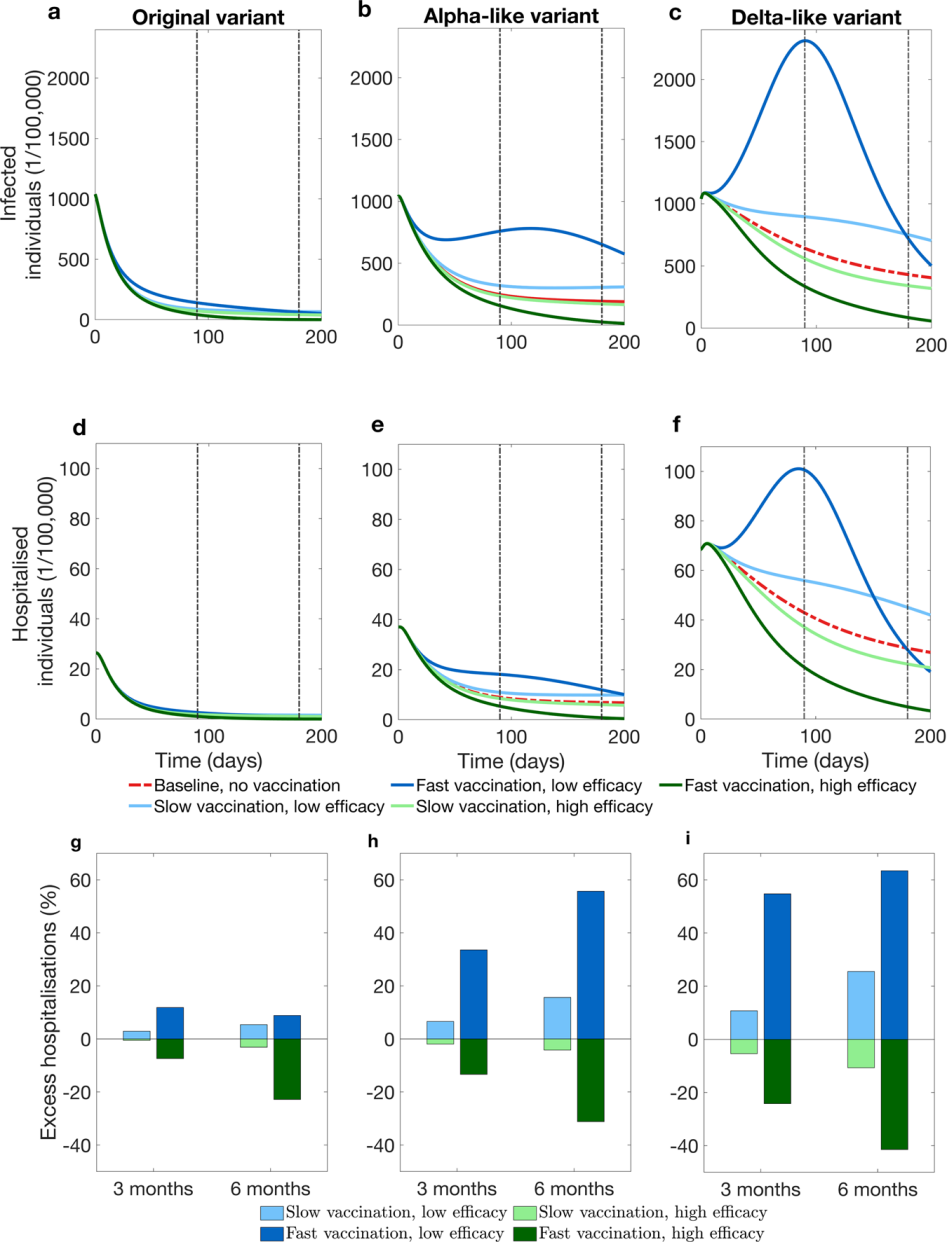
Epidemic dynamics with and without vaccination. **a** Prevalence of infected individuals versus time when the original variant circulates. **b** The same output when an Alpha-like variant circulates. **c** The same output when a Delta-like variant circulates. **d** Prevalence of hospitalised individuals versus time when the original variant circulates. **e** The same output when an Alpha-like variant circulates. **f** The same output when a Delta-like variant circulates. **g** Difference in the cumulative number of new hospitalisations relative to the no-vaccination scenario level for the original variant. **h** The same output when an Alpha-like variant circulates. **i** The same output when a Delta-like variant circulates. **g**–**i** show the difference in the cumulative number of new hospitalisations relative to the no-vaccination levels when respective variants circulate. Red curves on **a**–**c** correspond to no-vaccination scenario. Blue curves correspond to a vaccine with 60% efficacy in conferring protection against infection (low efficacy), green curves correspond to vaccine with 91% efficacy (high efficacy). In **a**–**f**, vertical brown lines mark three and 6 months since the start of vaccination.

The model predicts that depending on the speed of the vaccination rollout and transmissibility of the dominant virus variant, as a result of decreasing compliance with physical distancing measures, the prevalence of infected and hospitalised individuals in the presence of vaccination can be temporarily higher than the prevalence in a situation without vaccination (Fig. 3). Whether this occurs depends on a number of factors, for example, vaccine efficacy in conferring the immunity against infection acquisition. If a temporary increase in prevalence appears, it is more pronounced for the more transmissible Alpha-like and Delta-like variants than for the original variant (Fig. 3a–f).

If the original variant is circulating (Fig. 3a, d and g), vaccination can reduce both the prevalence of infected and the prevalence of hospitalised below the level of the no-vaccination scenario almost instantaneously (Fig. 3a and d, green curves) or it may take as much as 6 months after the start of the campaign (Fig. 3a, blue curves).

If the vaccine efficacy is high, the effect of the vaccination campaign is expected to be positive with prevalences of infected and hospitalised decreasing below the no-vaccination scenario almost immediately, such that larger reductions in the cumulative number of new hospitalisations relative to the no-vaccination scenario are expected for a faster vaccination rate (Fig. 3g, green bars).

Given a low vaccine efficacy, we observe a reverse situation for both vaccination rates due to the decline of compliance following the growing vaccination coverage. In this case, in the initial stages of the rollout, the transient prevalence of infected and hospitalised individuals can be higher than in the no-vaccination scenario and this increase is larger for faster vaccination rate (Fig. 3a and d, blue curves). Consequently, slow vaccination, if associated with decline of compliance during vaccine rollout, leads to a smaller excess of cumulative hospitalisations than fast vaccination at both three and 6 months time points (Fig. 3g, blue bars). However, on the positive side, we observed in our simulations that if vaccination rate is fast then the prevalence of infected cases decreases below the level of the no-vaccination scenario 6 months after the start of vaccination rollout. When vaccination rate is slow, the prevalence eventually decreases below the level of the no-vaccination scenario, but it can take >800 days (see Supplementary Fig. 20 in Supplementary notes).

If a more transmissible variant is circulating (for example, an Alpha-like or a Delta-like) (Fig. 3b, c, e, f, h and i) vaccination rollout can cause an almost immediate decrease in the cumulative number of new hospitalisations or decreased compliance with physical distancing measures can lead to an additional peak in prevalence of infected and hospitalised individuals (Fig. 3b and e). In this latter case, similar to the scenario with the original variant, if vaccine efficacy is low, vaccination can initially lead to an increase of cumulative number of new hospitalisations compared the no-vaccination scenario (Fig. 3h, i). This occurs because decline of compliance coincides with an increased transmissibility of the virus. The period when the prevalence of infected and hospitalised individuals is higher as compared to the no-vaccination scenario lasts even longer than for the original variant (Fig. 3a–f).

#### Contribution of vaccinated and non-vaccinated individuals to the attack rate

**Fig. 4:**
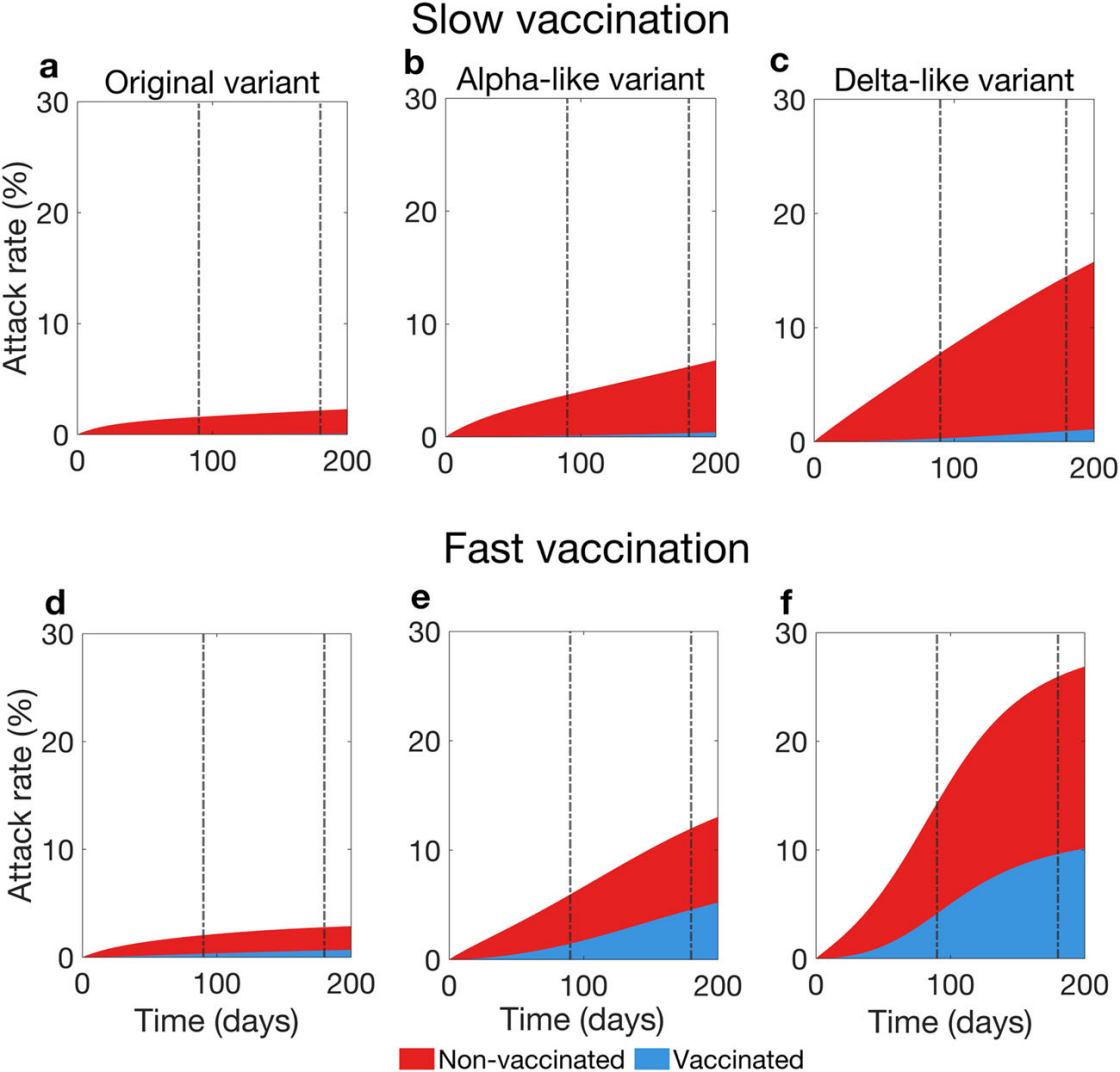
Contribution of vaccinated and non-vaccinated individuals to attack rate during the vaccination rollout. We consider the scenario with low vaccine efficacy in conferring immunity against acquisition of infection (60%). **a**–**c** show attack rates versus time given the slow vaccine uptake rate. **d**, **e**, and **f** show attack rates versus time given the fast vaccine uptake rate. **a** and **d** show these quantities for the original variant, **b** and **e** for an Alpha-like variant, **c** and **f** for a Delta-like variant. Vertical brown lines mark three and 6 months since the start of the vaccination campaign. Attack rate is the proportion of the population that has been infected until a given time. We adjusted the attack rate so that it describes only new infections that appeared during the time interval that we considered.

**Fig. 5:**
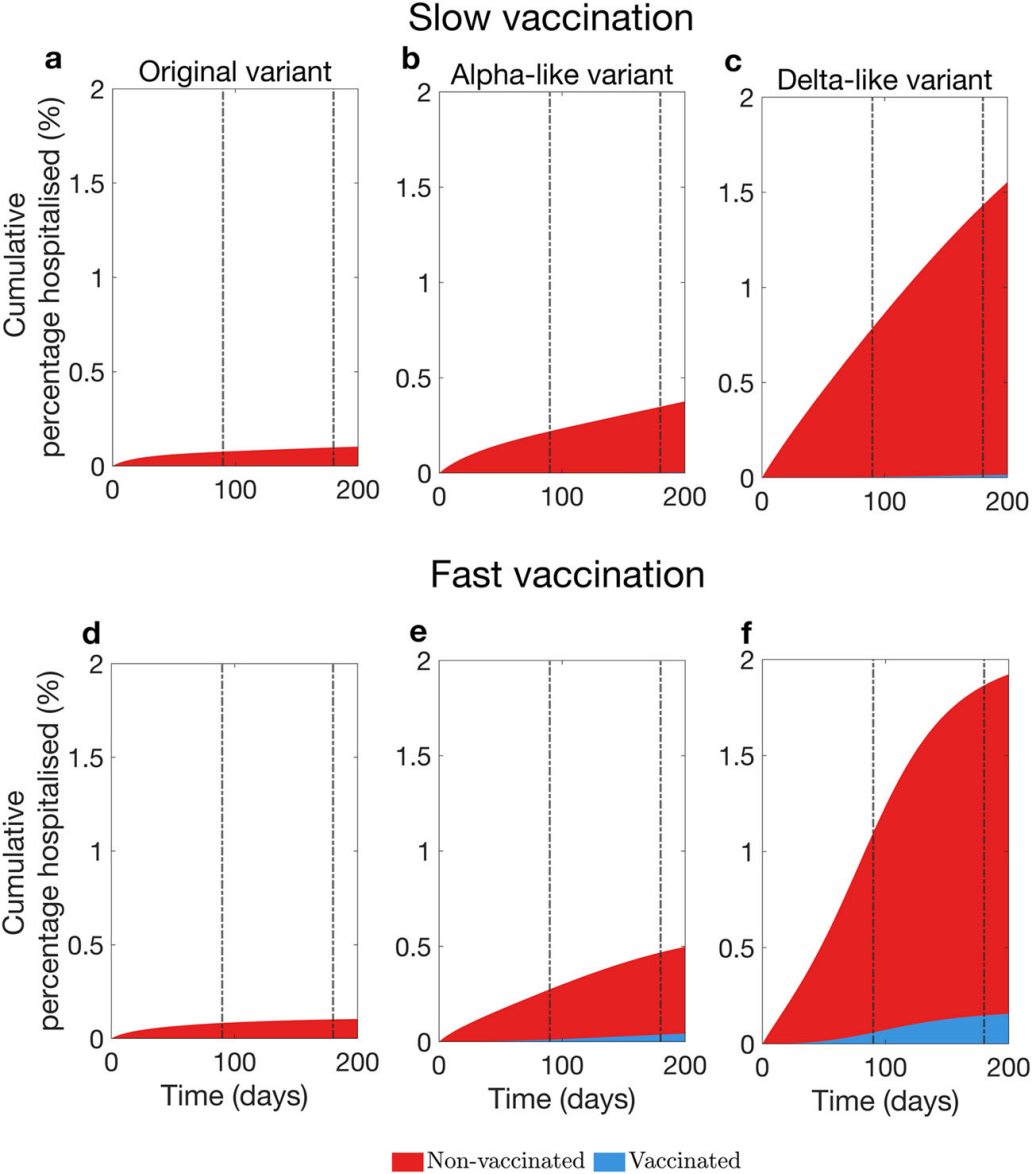
Contribution of vaccinated and non-vaccinated individuals to percentage of hospitalised individuals during the vaccination rollout. We consider the scenario with low vaccine efficacy in conferring immunity against acquisition of infection (60%). **a**–**c** show proportion of hospitalised population versus time given the slow vaccine uptake rate. **d**–**f** show the same output versus time given the fast vaccine uptake rate. **a** and **d** show this output for the original variant, **b** and **e** for an Alpha-like variant, **c** and **f** for a Delta-like variant. Vertical brown lines mark three and 6 months since the start of the vaccination campaign. We adjusted the percentage of hospitalised individuals so that it describes only new infections that appeared during the time interval that we considered.

We have seen that for a vaccine with low efficacy a transient increase in prevalence of infected and hospitalised individuals may appear. In what follows we investigate the role of vaccinated individuals in the transmission dynamics presented in Fig. 3 for vaccine with low efficacy. Re-call that the primary goal of COVID-19 vaccine was to reduce the probability of developing a severe disease as a result of becoming infected. Therefore, if a large proportion of infected population has been vaccinated, the temporary rise in prevalence may not necessary translate to a large increase in hospitalisation numbers. To identify conditions when this happens, we considered the proportion of infections occurring in the vaccinated population over time (Fig. 4) given low efficacy in conferring immunity against infection acquisition (60%). The analyses show that in the case of slow vaccine uptake (Fig. 4a–c) vaccinated individuals comprise a small proportion of the infected population even at the end of the 6 months of the vaccination campaign (Fig. 5a–c). Therefore, the increased prevalence among non-vaccinated can be attributed to the decrease of their compliance with physical distancing measures.

In the case of fast vaccine uptake, the model predicts that a proportion of infections among vaccinated individuals is higher. Moreover, for a “hyper-contagious” strain, similar to the Delta variant, more than a third of infections are expected to be in the vaccinated population. Thus, the observed rise in the prevalence is in part due to the increased contact rate of susceptible vaccinated individuals. These findings suggest that for slow vaccination the risk of severe disease and death in the population is hardly lowered, while for fast vaccination a considerable proportion of the infected individuals will be protected against severe disease, even if the incidence of cases is high (Fig. 5d–f). This proportion is higher for a more contagious strain.

#### Sensitivity of the vaccination rollout outcomes to vaccine efficacy and vaccine uptake rate

We have seen that depending on factors such as vaccine efficacy, the transient outcomes of vaccination campaign may be different. Therefore, it is important to investigate systematically the conditions in which a potential negative effects, such as temporary increase in the prevalence of hospitalised individuals above the no-vaccination level, can arise. In this section we present, the findings of the exploration of the joint effects of vaccine efficacy and vaccine uptake rate on the excess of the cumulative number of new hospitalisations as compared to the no-vaccination scenario three and 6 months after the start of vaccination rollout.

**Fig. 6:**
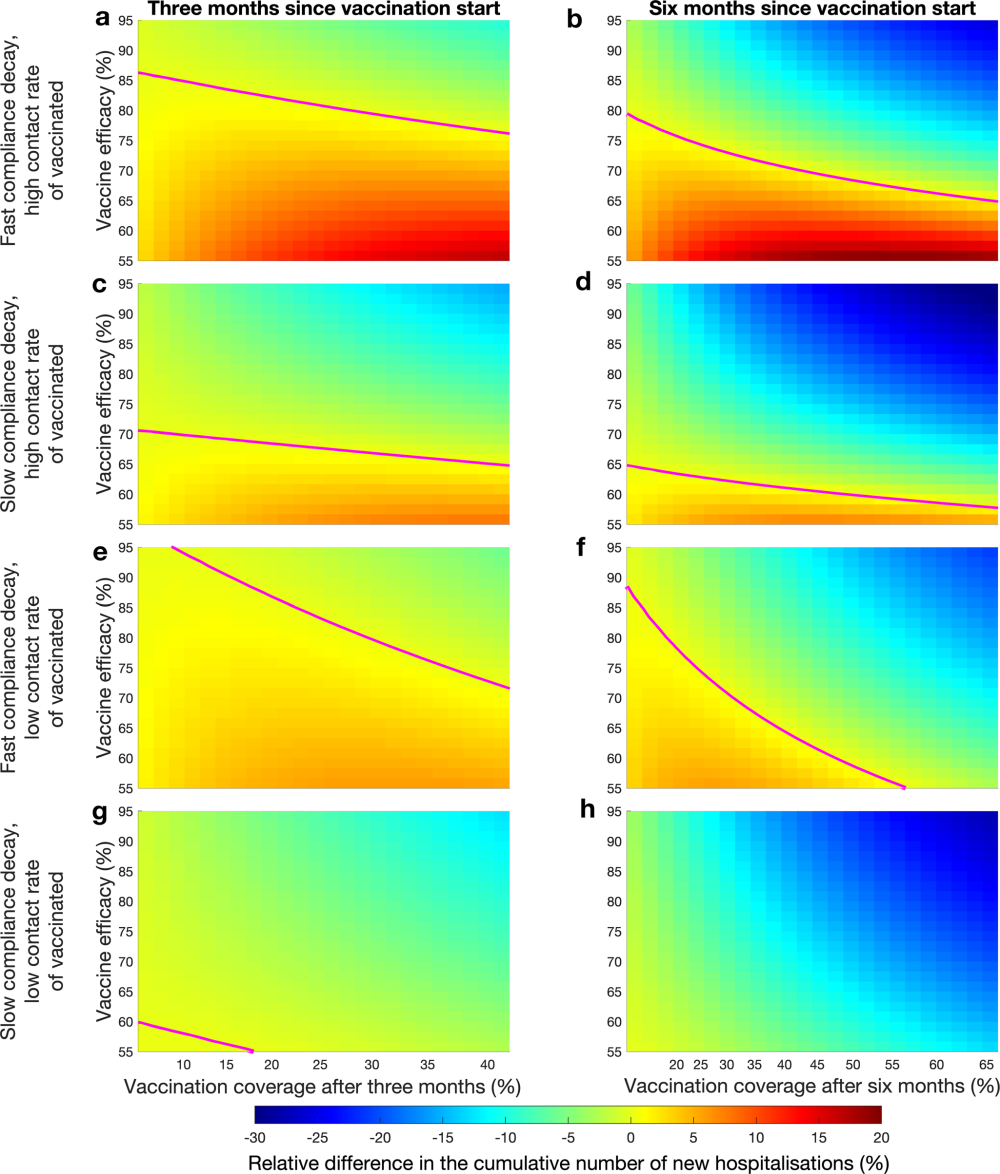
Epidemic dynamics with and without interventions targeting compliance of vaccinated and non-vaccinated individuals. The original variant of the virus circulates. All panels show relative difference in the cumulative number of new hospitalisations as compared to the no-vaccination scenario. **a**, **b** Vaccination rollout not supplemented with compliance interventions three and 6 months into the vaccination rollout, respectively. **c**, **d** Vaccination rollout supplemented with compliance interventions targeting non-vaccinated individuals three and 6 months into the vaccination rollout, respectively. **e**, **f** Vaccination rollout supplemented with compliance interventions targeting vaccinated individuals three and 6 months into the vaccination rollout, respectively. **g**, **h** Vaccination rollout supplemented with compliance interventions targeting both vaccinated and non-vaccinated individuals three and 6 months into the vaccination rollout, respectively. Magenta curves mark boundaries between parameter regions with different sign of the cumulative number of new hospitalisations. The scale of x-axis is not linear since the axes were obtained by conversion of the vaccine uptake rate to the vaccination coverage following three and six months after the start of the vaccination rollout.

Our results are summarized on Fig. 6a, b and Supplementary Figs. 5a, 5b, 6a, and 6b. In all panels, in the region above the magenta curve vaccination rollout yields improvement over the no-vaccination scenario, i.e. the cumulative number of new hospitalisations is lower. Importantly, the slower is the vaccination rollout, i.e. the lower is the vaccination coverage after 3 months of the vaccination rollout, the higher the vaccine efficacy needs to be to avoid an increase of cumulative number of new hospitalisations as compared to the no-vaccination scenario. This is a consequence of fast loss of compliance with physical distancing measures as the vaccination coverage grows. Vice-versa, depending on the vaccine efficacy, the speed of the rollout can cause increase or decrease of cumulative number of new hospitalisations. If the efficacy is low and the rollout is fast, then initially the cumulative number of new hospitalisations is expected to be higher than for the no-vaccination scenario. Moreover, the combination of fast vaccine uptake and low vaccine efficacy is predicted to cause the largest increase in the cumulative number of new hospitalisations as compared to the no-vaccination scenario. This happens due to the combined effect of quickly growing vaccination coverage which affects compliance with physical distancing measures in the non-vaccinated population and of increased contact rates of the vaccinated individuals who while potentially protected from the severe disease can still acquire and transmit the infection. However, if the vaccine efficacy is high, given a fast vaccination rate, we expect that the cumulative number of new hospitalisations to fall below the level of the no-vaccination scenario. The decrease in the number increases as the vaccination rate increases. We observe that for all variants considered (see Fig. 6a, b and Supplementary Figs. 5a, b, 6a, and b), the minimal vaccine efficacy where the cumulative number of new hospitalisations decreases over the no-vaccination scenario decreases with time since the start of the vaccination rollout.

We refer to the analyses above as to the epidemic dynamics without compliance-targeted interventions. In the following section, we investigated the impact of interventions targeted at maintaining compliance with physical distancing; we compared this to the epidemic dynamics without compliance-targeted interventions and the scenario without either vaccination or interventions.

### Interventions targeting compliance

To investigate how interventions may improve the impact of vaccination rollout, we considered an intervention that targets compliance of those who are not yet vaccinated and an intervention targeted at the vaccinated population. We assume that the first intervention targets non-vaccinated individuals and is successful in keeping the average duration of compliance at the pre-vaccination length (30 days) as vaccination coverage grows. The second intervention, targeted at vaccinated individuals, succeeds in convincing vaccinated individuals to abstain from increasing the contact rate above that of the contact rate of non-compliant individuals. Our model predicts that a successful implementation of either of these interventions reduces the cumulative number of new infections after vaccination rollout and can get this number below the level of the no-vaccination scenario. The effectiveness of these interventions depends on the circulating variant and the vaccine uptake rate. We summarize our findings in Fig. 6 and Supplementary Figs. 2–6.

#### **Intervention 1: targeting compliance of non-vaccinated individuals**

For all three variants, an intervention that targets compliance of non-vaccinated individuals (Fig. 6c, d and Supplementary Figs. 2c, 2d, 3c, and 3d), reduces the minimal efficacy of vaccine required for the cumulative number of new hospitalisations after three and six months following the vaccination rollout to be smaller than in the no-vaccination scenario at the respective time points. If there is an excess of new hospitalisations (area below the magenta curve), this excess is smaller than the excess number of the vaccination campaign not supplemented with the compliance-targeting intervention scenario (Fig. 6a, b, c, and d and Supplementary Figs. 2a–d, 3a–d).

#### **Intervention 2: targeting compliance of vaccinated individuals**

Effects of this intervention on the cumulative number of new hospitalisations depend on the circulating virus variant, vaccine efficacy, and vaccination rate (Fig. 6e, f and Supplementary Figs. 2e, 2f, 3e, and 3f).

For the original variant and a slow vaccination rate, we observe that after three months of vaccination for the whole range of vaccine efficacies that were considered there is excess of hospitalisations as compared to the no-vaccination scenario (Fig. 6e). This is contrary to the scenario when the vaccination rollout is not supplemented with compliance-targeted interventions, where vaccinated individuals are characterized by the increased contact rate. This outcome occurs due to the change in mixing. As vaccinated individuals have less contacts, more transmission contacts occur in the non-vaccinated population leading to the increase in the number of infections. Behavioural research in the context of COVID-19 conducted in the Netherlands in November and December 2021^70^ showed such contact transfer to be a possible outcome.

If the vaccination rate is fast, three months after the start of the vaccination rollout, we also see mixed results. For the combination of the vaccine efficacy and vaccination rate which gives a decrease in the cumulative number of the new infections in the scenario where vaccination rollout is not supplemented with compliance-targeted intervention, we see this decrease reducing in magnitude (the upper right corners of Fig. 6a and e). On the other hand, the minimum of vaccine efficacy where the cumulative number of new hospitalisations is below the no-vaccination level is lower than in scenario where vaccination rollout is not supplemented with compliance-targeting intervention. Finally, the region with excess infections in the lower right-corner is lighter shade indicating smaller number of new hospitalisations compared to the scenario where the vaccination rollout is not supplemented with compliance-targeted interventions.

Six months after start of the vaccination rollout, the situation is similar (Fig. 6f). Given a slow vaccination rate, the minimum of vaccine efficacy where the relative increase of hospitalisations can be avoided is higher than in the scenario where the vaccination rollout is not supplemented with the intervention. But if the vaccination rate is fast, than the respective vaccine efficacy minimum is lower than it was without the intervention.

The dynamics for different regions of the vaccine efficacy and vaccination rate for an Alpha-like or a Delta-like variants when the intervention is deployed are qualitatively similar to the dynamics of the original strain (Supplementary Figs. 5e, f, 6e, and f).

#### **Combination of two interventions**

Finally, combination of the two compliance-targeting interventions leads to improvements that exceed the effects of individual interventions (Fig. 6g, h and Supplementary Figs. 5g, 5h, 6g, and 6h). For all three variants, the minimum for vaccine efficacy where the excess of infections as compared to the scenario without compliance-targeted intervention can be avoided, is dexcreased. Also, excess in the cumulative number of hospitalisations is decreased for the region of vaccine uptake rate and vaccine efficacy that we considered. Similar reductions relative to the scenario where the vaccination rollout is not supplemented with compliance-targeting intervention are achieved for the more transmissible variants, provided a fast vaccination rate (Supplementary Figs. 5g, 5h, 6g, and 6h).

#### **Supplementing vaccination rollout with a lockdown**

Our simulations indicated that due to compliance declining as the vaccination coverage grows, it is possible that an additional prevalence peak appears. So far, in our simulations no centralized intervention triggered by a steep increase in the number of new cases was modelled. Here we consider such an intervention, whereupon if during the vaccination rollout the prevalence of new infectious cases exceeds a certain threshold, the government tightens the lockdown, further restricting the average contact rate, including vaccinated individuals. Once the prevalence falls below the threshold, the lockdown is being relaxed to its prior state. We investigated the effect of the threshold prevalence at which the lockdown is initiated on the cumulative number of new infections three and six months after the start of the vaccination campaign (Supplementary Figs. 7–9). We considered vaccine efficacy and vaccination rate on the ranges used for the main analysis.

Our simulations indicate that supplementing the vaccination rollout with lockdown which initiates once the prevalence of infectious cases exceeds a threshold can prevent increase of the cumulative number of new infections after three and 6 months of the vaccination rollout as compared to no-vaccination scenario (Supplementary Fig. 7). The cumulative number of new infections after three months of the vaccination rollout are larger for the fast vaccination rollout than for the slow. Interestingly, the cumulative number after three months of the vaccination rollout for either vaccination rate is not sensitive to changes in the lockdown strengthening/relaxation threshold on the range that we consider. On the other hand, the cumulative number of new infections after six months of the vaccination rollout for both slow and fast vaccination rates is increasing as the threshold for the strengthening/relaxation of the lockdown grows. While the relative difference in the cumulative number of new infections is larger for a lower lockdown initiation threshold, the difference for the extrema of lockdown initiation threshold is below 10%. The largest decrease in the cumulative number of new infections relative to the no-vaccination scenario happens when the vaccination rate is fast and the vaccine efficacy is high. Decreasing either one of these parameters causes the relative difference to decrease (Supplementary Fig. 8). On the other hand, the largest decrease in the cumulative number of new infections relative to the vaccination rollout without compliance interventions happens when the vaccination rollout is fast and the vaccine efficacy is low (Supplementary Fig. 9).

This concludes our analysis of the sensitivity of epidemic dynamics to the key model parameters. To see the complete sensitivity analysis of the model with respect to the rest of the parameters, see Supplementary notes, Figs. 14–19.

## Discussion

Using a compartmental model for the spread of SARS-CoV-2 in a population, where physical distancing measures are in place, we investigated the impact of declining compliance with physical distancing measures as vaccination is rolled out on the numbers of infections. One of the key features of our model is a distinct treatment of the loss of compliance by vaccinated and non-vaccinated populations, each of which can relax the compliance of physical distancing measures to a different degree. Additionally, we extended the compliance process to the whole population and not only the susceptible individuals, which qualitatively affects mixing patterns in the population.

Our main finding is that, if compliance decays as the vaccination coverage grows, the speed of vaccination rollout has a strong impact on whether the cumulative number of new infections can be decreased three and six months after the start of vaccination below the level that would have been expected without vaccination (Fig. 3). The outcome will depend on the vaccine efficacy in conferring protection against infection. If vaccine efficacy is low, it may lead to an increase in the prevalence exceeding the prevalence in a situation without vaccination and, in the short term, we may even see an additional epidemic peak. Moreover, in the transient stages of the rollout, worse outcomes can be expected for a faster vaccination rate. This effect happens due to the loss of compliance by vaccinated individuals. On the other hand, if vaccine efficacy is relatively high, these detrimental effects can be avoided. Moreover, the decrease in prevalence will be larger for faster vaccination rates. However, we note that the available real world data suggests that even in the case when vaccine has a high efficacy, if the physical distancing measures during the initial period of the rollout are relaxed below a threshold, a new wave of infections may happen^71^. The authors of this study analysed the data on vaccinations and COVID-19 incidence collected in Cyprus and Malta between December 2020 and June 2021 during vaccination rollout and observed that Cyprus which supplemented vaccination rollout with strict public distancing measures saw a decline in new cases, despite its vaccination uptake rate being slower than in Malta. Malta, on the other hand, experienced a sharp uptick of new infections in the first stages of vaccination campaign due to more relaxed public distancing measures. Note that should there be an increase in infections relative to the no-vaccination scenario, since among the excess infections a certain proportion of infected people will have been vaccinated, they will have a low probability of developing severe disease or death.

Finally, as a result of our comprehensive analysis of the effect of the vaccination rate and vaccine efficacy on the cumulative number of new infections and new hospitalisations, we derived threshold curves which separate parametric regions where the relative difference in the cumulative number of hospitalisations as compared to the no-vaccination scenario changes sign (Fig. 6). We observed, that if the vaccine has a high efficacy, then the excess of hospitalisations can be avoided for a relatively low vaccination uptake rate. As the vaccine efficacy decreases, the uptake rate increases.

In their recently published work Gozzi et al^72^ also considered the impact of the feedback between the epidemic dynamics, the vaccination rollout, and compliance with physical distancing on infection transmission dynamics. The authors investigated the effects of declining compliance due to the growing vaccination coverage provided different vaccination strategies and vaccine efficacies across populations with different age contact matrices. Both ours and Gozzi et al^72^ qualitative findings are in agreement and are consistent with the results of the earlier studies that have shown that factors that contribute to drastic increase of contact rates (such as vaccination-related behavioural change or premature reduction/removal of non-pharmaceutical interventions) may reduce the benefits of a vaccination programme^73–76^.

Motivated by the conclusions drawn by these studies, we considered the effect of supplementing the vaccination campaigns with communication strategies promoting maintenance of physical distancing behaviour aimed at both vaccinated and non-vaccinated individuals and learned that (1) those interventions can substantially improve the outcome of vaccination campaign; (2) the choice of a specific information intervention should be informed by the epidemic circumstance of the situation (such as the dominant variant and speed of vaccination rollout).

An intervention that succeeds in maintaining the compliance with physical distancing in people not yet vaccinated on the same level as before the start of vaccination ensures substantial decrease of the cumulative number of new infections and subsequently hospitalisations and deaths throughout. Moreover, we observed that for all three virus variants that we considered, supplementing vaccination rollout with this intervention reduces the vaccine efficacy threshold for which the cumulative number of new infections is lower than without the vaccination. This effect is seen in both short and long term, but is more pronounced in the long term. The effect for an intervention that targets vaccinated individuals to prevent them from increasing their contact rates after being vaccinated depends on the transmissibility of the dominant variant. If the original variant circulates, the intervention has a positive impact for a fast rollout of vaccination, but cannot avoid detrimental effects of decline of compliance if the vaccination rollout is slow. On the other hand, if the dominating variant has the same transmissibility as Alpha or Delta, then the intervention can improve the outcome of the vaccination rollout over the no-vaccination scenario even when the vaccination rate is slow. Interestingly, for the original and an Alpha-like variant, given a slow vaccination rate, the minimum vaccine efficacy threshold required to avoid a surplus of infections is higher when the vaccination rollout is supplemented with the intervention than when it is not. If a Delta-like variant circulates, supplementing the vaccination rollout with the intervention reduces the threshold for all vaccination rates that we considered. Only the combined effect of both interventions can consistently reduce the cumulative number of new infections below the level of the no-vaccination scenario regardless of the rollout speed (in the vaccination rate range that we considered).

Finally, we compared the effect of compliance-targeting interventions with an intervention that mimics tightening/relaxation of the lockdown when a prevalence threshold is crossed. We observed that on its own the lockdown intervention eliminates the possibility of an excess of infections and yields larger decreases in the cumulative number of new infections over the no-vaccination scenario than the compliance-targeting interventions, both in the short term and in the long term. The outcomes of supplementing the rollout with this intervention are not sensitive to the prevalence threshold. However, it may come at a price of disrupted social fabric and slowing down of the economy.

Our results are based on some simplifying assumptions, one of them that physical distancing measures remain in place throughout the time period of analysis (6 months). While this would be advantageous for preventing transmission of the virus, it might not be feasible out of societal and economic reasons. Therefore, compliance rates may wane even faster in real populations and contact rates may be up to higher, possibly pre-pandemic values during the rollout of vaccination. We do not expect that this would change our results much, as our results are obtained relative to the no-vaccination scenario, which would similarly be affected by a change in physical distancing measures. We expect therefore that the relative effects of vaccination would remain similar as in our simulations. We also assumed that the speed of vaccination rollout stays constant over the time period of 6 months, which is not the case in reality. In the Netherlands for example, vaccination rates have increased substantially after a slow start in January 2021^42^. These rates will depend on many factors, nevertheless large differences will remain between countries. Finally, we have captured the dependence of rates of becoming compliant and non-compliant on the incidence of new infectious cases and vaccination coverage, respectively, using linear functions. As the vaccination in many countries continues and the population response data is collected, a more precise formulation of the response functions can be obtained. However, our results predominantly depend on the assumed monotonicity of these functions.

Furthermore, our model is relatively simple, not taking into account age structure and heterogeneity in contact patterns. Therefore, we do not attempt to make quantitative predictions on the impact of vaccination, but we provide qualitative insight into possible effects of decline of compliance with physical distancing in the face of increasing vaccination coverage. Since the time of our conceiving and completing this study a new variant of concern, Omicron, has emerged^77,78^, whose hallmark is escaping natural and vaccine-induced immunity. There is evidence that this variant is characterized by increased transmissibility (as compared to previously dominant variants of concern). There is also evidence that infection with this variant is associated with reduced morbidity and mortality^77^, although there are indications that existing vaccination coverage plays a role in this observed reduction of severity. The findings of our study indicate that in the regime where Omicron is the dominant variant, the undesirable outcomes of the reduced compliance with physical distancing measures such as increased number of new infections can become further amplified, since the number of vaccinated individuals who can asymptomatically spread the infection would be even higher than for the Alpha or the Delta variants. Subsequently, given high transmissibility of the virus, a substantial number of hospitalisations and deaths in both non-vaccinated and vaccinated individuals can accrue.

A number of studies/reports estimated the bounds for vaccine efficacy for the original variant in terms of reducing the infection for some vaccines approved for use in Europe^24,25,79,80^. As Alpha (B.1.1.7) and Delta (B.1.617.2) variants emerged and, in turn, became dominant in many European countries, the first estimates for vaccine efficacy for reducing the infection became available^31,32,81^. Whether the reduction in infection comes in the guise of reduction of susceptibility or transmissibility of vaccinated individuals is not known. Therefore, in this work we modelled the vaccination to be all-or-nothing and vaccine efficacy was given in terms of probability of conferring full protection from becoming infected. Our sensitivity analyses (Fig. 6 and Supplementary Figs. 2–6) show that the effect of a vaccination campaign and of individual interventions is highly sensitive with respect to this parameter. However, we observed that if no compliance-targeting interventions accompany the vaccination rollout, the range of efficacies for which a surplus of new infections as compared to no-vaccination is possible three and six months following the vaccination rollout falls within the vaccine efficacy boundaries that were reported for different vaccines^24–27,29,31,32,80,82^. To implement the most efficient vaccination rollout it is important to know the boundaries of vaccine-conferred reduction of transmission.

Finally, in this work we have considered dynamics of circulation of three SARS-CoV-2 virus variants, the original variant and two mutations, whose transmission potential is similar to the Alpha and Delta variants. For all three variants, we modelled the immunity induced by the vaccine to be of the identical type (sterilising).

Our results also show that speed of rollout of a vaccination campaign is important, because the speed of the rollout and subsequent changes in contact rates strongly impact cumulative number of new infections. Although given the scenario where vaccine efficacy is low and vaccination rollout is fast the population may observe a higher number of new infections than it would have been without vaccination in the short term—especially for a more transmissible virus variant—on the longer term (>1 year) it has vast advantages in terms of numbers of infections prevented.

Our results emphasize the importance of communication by public health professionals on continued adherence to self-imposed measures, to those who are awaiting vaccination as well as to those already vaccinated. Communication messages need to be different and targeted specifically to these two groups. We highlight the positive overall effects of vaccination campaigns in combination with continued adherence to non-pharmaceutical preventive measures.

## Supplementary information

Supplementary information

Reporting Summary

## Data Availability

The datasets analysed during the current study are available in the GitHub repository for COVID-19 by Our World in Data website, https://github.com/owid/covid-19-data/tree/master/public/data. Additionally, the repository that contains the model code (https://github.com/aiteslya/VaccineCompliance1.2), includes a folder (FiguresData) with source data which can be used to produce Figs. 2–6 directly without needing to run the code itself. The data is stored in .csv format.

## Code availability

The model was implemented in MATLAB R2020b^83^. The code producing the analyses and figures for this study is available at https://github.com/aiteslya/VaccineCompliance1.2^64^.
